# Evaluating the illumination performance of LED fishing lamps with different configurations in Pacific saury fishery

**DOI:** 10.1371/journal.pone.0328676

**Published:** 2025-08-05

**Authors:** Fei Li, Chuanxiang Hua, Qingcheng Zhu

**Affiliations:** 1 College of Marine Living Resource Sciences and Management, Shanghai Ocean University, Shanghai, China; 2 National Engineering Research Center for Oceanic Fisheries, Shanghai, China; International Iberian Nanotechnology Laboratory, PORTUGAL

## Abstract

The configurations of artificial light on fishing vessels are important factors that affect underwater lighting characteristics, fish behavior, and catch efficiency in light fishery. A validated illumination model was used to investigate the illumination performances among three different shapes and mounting modes of commercial light-emitting diode (LED) fishing devices installed at angles of 45°, 60°, and 75°, respectively. The results showed that the illumination distributions of all these differently shaped or mounted LED modules exhibited a series of concentric iso-illuminance curves, but significant differences were found in the detailed lighting performances of the modules with different allocations. With increasing angle, the illuminance differences among the three types of LED modules decreased, and the deviations in the illumination distributions at different angles were mainly concentrated around the light source. Notably, at the same angle, the maximum illuminance value of a rectangular vertically mounted lamp (RVL) was always higher than those of the other two kinds of modules. The findings indicated that the LED fishing lamps manufactured into rectangular shape and mounted vertically at installation angles of 60° to 75° had superior effects in attracting saury.

## Introduction

The application of artificial light technology in fisheries has emerged as a highly advanced and practical approach for capturing economically fish species such as squid [1], Pacific saury [2], mackerel [3], and anchovy [4]. With growing concerns about energy shortages and global calls for carbon neutrality, light-emitting diode (LED) fishing lamps, well known for their low power consumption, high brightness, and long operating lifetime, have attracted worldwide attention from offshore and pelagic fishermen as an alternative to traditional light sources [5–7]. Due to the inherent limitations of individual LED chips in terms of power output and light intensity, commercial LED devices are manufactured in the form of packaged modules with integrated lamp bead arrays [8,9]. The arrangement of these beads plays a critical role in determining the module shape and how the modules are mounted on fishing vessels, which in turn affect the configuration of the onboard lighting system, the characteristic of the underwater light distribution, and the aggregation of fish shoals [10,11].

The Pacific saury (hereafter saury), a small pelagic and positively phototropic fish, exhibits a diurnal vertical migration pattern and inhabits water depths of 15–20 m [12,13]. Saury is widely distributed throughout the Northwestern Pacific Ocean and mainly harvested by fishing vessels from Japan, China, Chinese Taipei, Russia, South Korea, and Vanuatu [14]. From ancient torches to the incandescent bulbs of decades ago to modern LED lights, fishermen have used overwater light sources to catch saury during moonless nights for hundreds of years [15,16]. While the catch performance of LED light sources varies across different fisheries [17–19], commercial saury fishing activities over the decades have demonstrated that LED lamps outperform incandescent bulbs in terms of fuel savings and total catch yields [20,21]. The responses of fish aggregations and swimming behaviors to light stimuli, as well as the lighting characteristics of the underwater illuminated field, depend strongly on the configurations of the artificial lights [22,23]. However, it is important to recognize that there are significant differences in the shape and installation of LED fishing lamps that are utilized on saury fishing vessels in different countries and regions. For example, the LED modules in both China and Japan are rectangular in shape, but in China they are screwed vertically to the lamp poles (i.e., rectangular vertically mounted lamp, RVL) [2], while in Japan they are installed horizontally (rectangular horizontally mounted lamp, RHL) [21]. In contrast, the fishing lamps in Chinese Taipei are manufactured in circular shapes (circular lamp, CL), but there is no consensus on which module type is more attractive to saury. On the other hand, selection of the optimal mounting mode for fishing lamps is important to the availability of lighting and greatly affects the success or failure of fishing operations [24]. Typically, multiple LED modules are mounted or screwed onto rotating lamp poles, which are then attached to the sides of saury fishing vessels. However, the arrangement of these lamp poles (e.g., inclination angle, height, and spacing) tends to be based on personal experience or available deck infrastructure, which is neither scientific nor conducive to effective fish attraction [25].

Simulations of fishing light fields are an efficient method to understand underwater illumination characteristics, evaluate the lighting performance of fishing lamp systems, and design novel light sources. Zhu et al. formulated a theoretical illumination equation for incandescent bulbs by fitting experimental data obtained from saury fishing and investigated the illumination and light intensity of a single bulb when placed at the top, middle, and bottom of a light box installed at a 45° angle [26]. Based on the same illumination model, Hua et al. further carried out several sets of optical experiments on incandescent lamps to compare the distribution and variability of illumination at angles of 30°, 45°, and 60° [27]. A simulation of the illumination distribution around a fishing vessel was performed by Lai et al., who employed DIALux lighting software (DIAL GmbH, Lüdenscheid, Germany) to design and optimize a multisegmented freeform secondary lens for high-power LED fishing lamps [28]. Takahashi et al. presented a light distribution simulator aimed at investigating the irradiance distribution of LED fishing lights [29]. Hua et al. put forward a spherical light source approach to simulate the illumination distribution of an incandescent bulb [30]. According to the optical properties of individual LED lamp bead, Li et al. proposed an illumination model and described the illumination and light intensity distribution of a single fishing module [2]. Wang et al. developed a new computing model of underwater illumination of fishing lamp based on Monte Carlo method by using spectrum and luminous intensity of lamps and the inherent optical properties of seawater [31]. Choi evaluated the underwater irradiance within 30 m depth based on underwater spectral measurements [32]. Zhang et al. investigated the optimal configuration and three-dimensional light field of fishing lights on a squid fishing boat based on a spatial matrix simulation approach [33].

The important factors affecting the catch performance of the artificial light itself in attracting and gathering target fish include the type of light sources, the number of fishing modules, and the arrangement of the lamp poles. For a long time, fishermen believed that using powerful fishing lamps was necessary to achieve high yields, resulting in severe competition for high power light sources and consequently excessive fishing capacity [25]. In fact, the volume of illuminated water dose not simply increase proportionally with a continuous increase in the power or number of fishing lights [34]. To date, many efforts have been made to promote the use of reasonable numbers of fishing lamps and regulations to limit the total power of each fishing vessel [35]. However, limited research has been undertaken to evaluate the allocation of fishing lights to fish that prefer aggregation, and the scientific basis for determining the optimal shape and installation mode of LED fishing modules remains unclear. The aims of this study were to analyze the illumination performances of three types of fishing modules at different installation angles and to evaluate the optimal design of the module shape and arrangement of onboard fishing lights.

## Materials and methods

### LED fishing module

**Fig 1:**
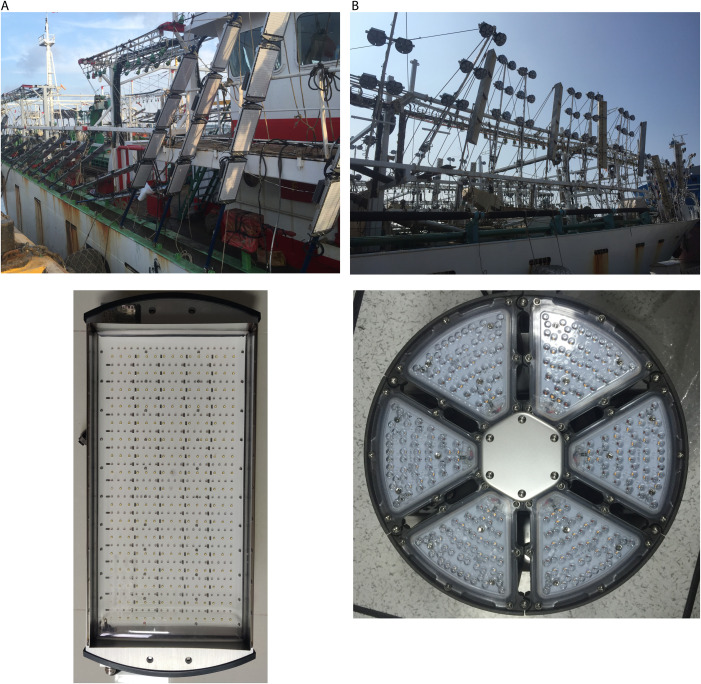
LED modules installations on Chinese saury fishing vessels on-site. **(a)** Rectangular vertically mounted lamp (RVL) made in China and **(b)** circular lamp (CL) made in Chinese Taipei.

Two types of LED modules (RVL and CL) that were mounted on Chinese saury fishing vessels are shown in Fig 1, where the RVLs are manufactured by Guangdi Photoelectric Science and Technology Co., Ltd. (4 modules attached to a lamp pole, each module with a power of 500 W), and the CLs are manufactured by Taipei Hushun Technology Co., Ltd. (6 modules attached to a pole, 312 W per module). The total power of a single lamp pole for both lamp types is approximately 2000 W.

**Fig 2:**
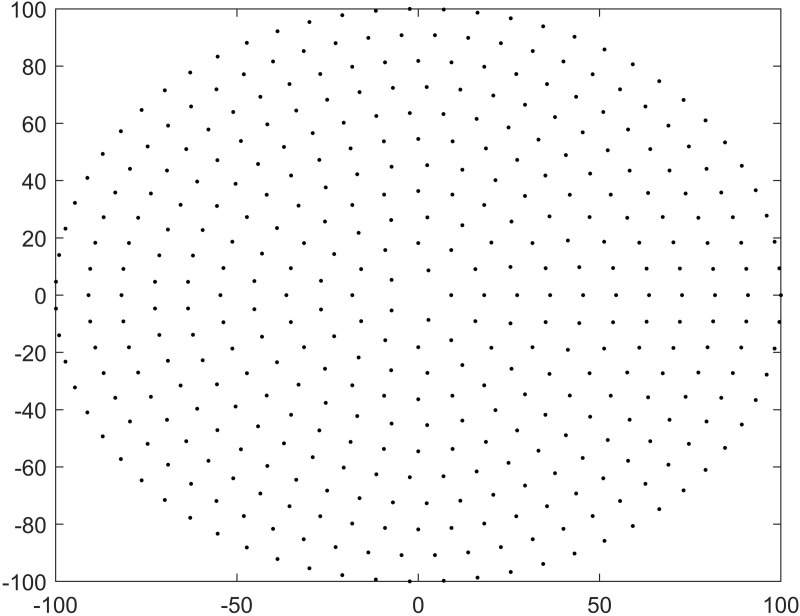
Design of the lamp beads in the virtual circular module (unit: cm).

To confirm whether the discrepancies observed in the lighting characteristics of various LED fishing modules are attributable to differences in their shape or arrangement, first, the rectangular module and the CL should have the same number of packaged lamp beads and effective illumination area as possible for comparison. Second, since it is very difficult to measure and determine the exact positions of the irregularly arranged lamp beads in the CL (Fig 1(b)), a similar virtual CL with uniformly arranged lamp beads was designed (Fig 2) based on the Chinese rectangular-shaped LED module (Table 1). The number of lamp beads and the working electric power are the same for both rectangular and circular modules.

**Table 1:** Parameters of the rectangular LED module (unit: cm).

Module length	Module width	Horizontal column	Horizontal spacing	Vertical column	Vertical spacing
70	31	20	1.18	20	2.58

### Mathematical model

A theoretical illumination model for the LED module has been proposed by combining the optical properties of each lamp bead and the superposition properties of the illumination, which enables calculations of the illumination by onboard fishing lamps at any point in the illuminated surface. Details on the optical experiments of the lamp beads, the construction of the illumination model and its validation are available in our previous work [2]. In this section, only a brief description of the illumination modeling process is provided and uses the rectangular module as an example.

**Fig 3:**
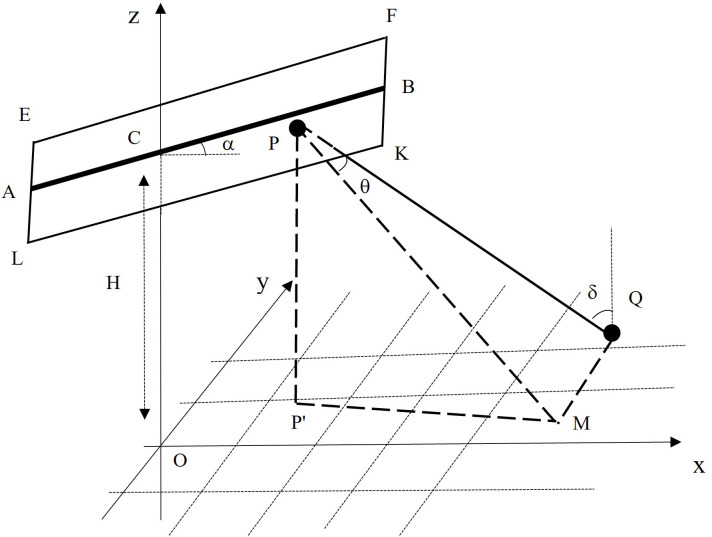
Schematic diagram of the spatial relationships between the module and the illuminated area.

The spatial relationships between the lamp beads in the module and an arbitrary position in the illuminated area are depicted in Fig 3. EFKL represents a rectangular vertically mounted LED module, AB is the axis passing longitudinally through the center of the module, C is the midpoint of AB, and O is the vertical projection point of C in the illuminated area. Point O is set at the origin of a Cartesian coordinate system, the *x*-axis is the projection of AB in the illuminated area, the *y*-axis is the projection that passes transversely through C and is perpendicular to the *x*-axis, and the positive *z*-axis is upwards. H is the vertical height from C to the illuminated area, P is an arbitrary lamp bead in the module, and P’ is the vertical projection of P in the illuminated area. M is the intersection of the output beam in the direction of the optical axis of the bead and the illuminated area, and Q is an arbitrary point in the illuminated area. *α* is the angle between AB and the horizontal direction (i.e., the inclination angle of the module), *θ* is the angle between the optical axis and PQ, and *δ* is the angle between PQ and the normal at Q.

**Fig 4:**
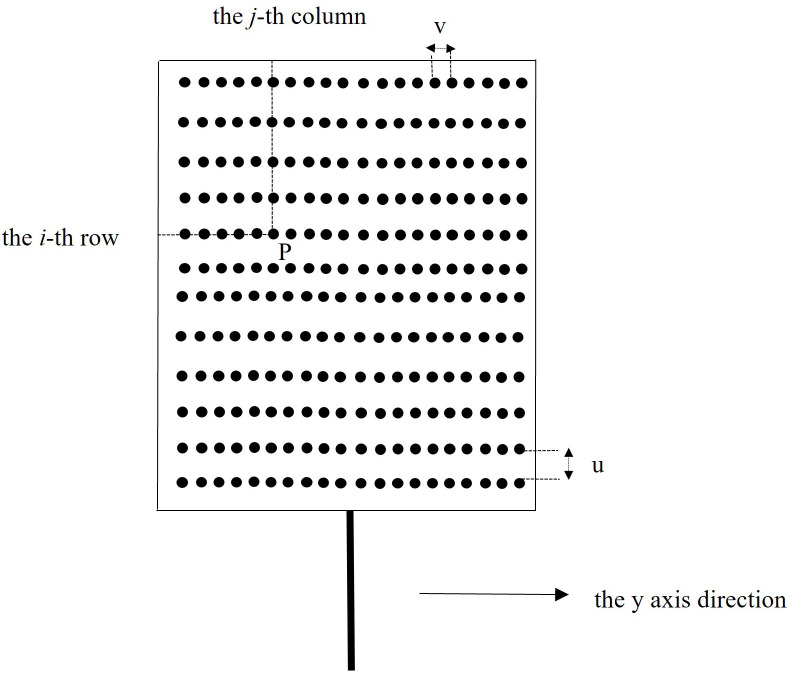
Schematic arrangement of lamp beads in the rectangular-shaped LED module.

The rectangular-shaped LED module shown in Fig 4 consists of an array of 400 lamp beads arranged in 20 rows and 20 columns. Assume that *P* (*i*, *j*) is an arbitrary single bead that is located in the *i*-th row from bottom to top and the *j*-th column from left to right in the module. *u* and *v* are the distances between two adjacent beads in the same row and column, respectively.

The light distribution of an LED lamp bead is a multiple cosine function of the scattering angle, and the light intensity can be described as follows:


$$I_{\theta }\;=\;I_{0}\cdot \cos^{m}\theta$$ (1)

Where *θ* is the scattering angle between the optical axis and an arbitrary light beam (Fig. 3), *I*_θ_ is the light intensity of the LED bead in the direction of *θ*, and *I*_0_ is the light intensity in the normal direction of the bead. The constant *m* is the Lambertian mode number, which refers to an optical parameter related to the scattering angle of the light source and can be calculated by fitting the light intensity data of the bead in different directions in space. In this study, the value of the optical parameters *I*_0_ and *m* for the LED bead were 15.1 cd and 0.79, respectively.

A single LED lamp bead can be considered a point light source [36], which means that its luminous intensity and distance can be described by the square inverse law. The illumination E_*PQ*_ of the bead at any point Q in the illuminated area is given by:


$$E_{PQ}\;=\;\frac{I_{\theta }\cdot \cos\delta }{PQ^{2}}$$ (2)

Where E_*PQ*_ is the illumination from P to point Q in the illuminated area, *δ* is the angle between PQ and the normal to Q, and PQ is the distance between P and point Q.

According to the cosine formula and trigonometric function transformation, *θ* can be obtained by:


$$\theta \;=\;\arccos\frac{PQ^{2}+PM^{2}-QM^{2}}{2PQ\cdot PM}$$ (3)

Where PQ, PM, and QM are the distances between P and Q, P and M, and Q and M, respectively.

The illumination E_*PQ*_ of the bead at any point Q in the illuminated area is represented as:


$$E_{PQ}\;=\;\frac{I_{0}\cdot \cos^{m}\theta \cdot Z_{P}}{PQ^{3}}$$ (4)

Under the assumption of incoherence of the optical properties among different lamp beads, the sum of the illuminance values of 400 LED beads at any point Q in the illuminated area is obtained by:


$$E_{-total}=\sum_{i=1}^{20}\sum_{j=1}^{20}E_{PQ}$$ (5)

Where *E*__*total*_ is the total illumination of one LED module at any point in the illuminated area.

In the present study, there are 400 individual lamp beads within a fishing module, and it is assumed that there are no significant differences in the optical properties of all the beads. However, for the onboard lighting system consisting of more than four hundred LED modules (i.e., at least 160000 lamp beads), illuminance calculations for different configurations of fishing lamp at any given location would be intolerable with current computing capacity. Considering that the main purpose of this work is to compare which kind of lamp shape and installation angle achieves the optimal illuminance characteristics, only a single fishing lamp was studied.

### Data processing and analysis

The illuminance values of an RVL, RHL, or CL installed at 45°, 60°, and 75° were predicted by the above illumination model, and the differences in illuminance between the RVL and the other two fishing modules were simulated by MATLAB software (v. R2022a, The MathWorks, Inc., Natick, America), respectively. To obtain more detailed information about the lighting characteristics (e.g., illumination distributions and corresponding lighting attenuation) of the three types of LED fishing lamps at different angles, the variations in illuminance values and attenuation rates along the *x*-axis as well as the illumination curves of 10 and 1000 lx were explored.

## Results

### Illumination characteristics of fishing lamps with different shapes

#### The rectangular vertically mounted lamp (RVL).

**Fig 5:**
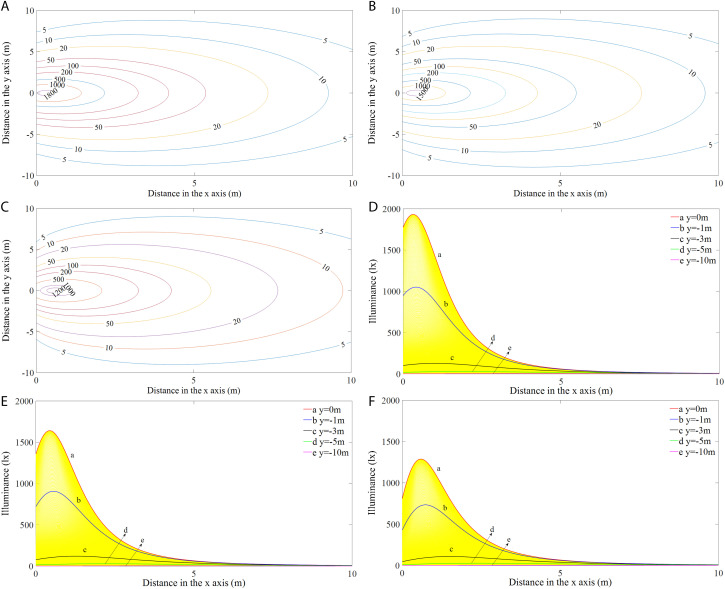
Illumination distributions (a-c) and variations in illuminance values along the *x*-axis (d-f) of the RVL at different angles.

Fig 5 shows the simulated illumination distributions (left) and the corresponding variations in illuminance values at *y* = 0, −1, −3, −5, and −10 m along the positive *x*-axis direction (right) of the RVL at different installation angles. As presented in Fig 5(a-c), the iso-illuminance curves form a series of concentric circles with the light intensity distributions being symmetrical to the axis *y *= 0 m in the light field. The illumination levels in the region that was closer to the LED module were higher and more variable than those farther away and generally decreased with increasing distance. The maximum illuminance value was not observed at the origin but at a position on the axis in the direction of *y *= 0 m. More specifically, as seen from curves a and b (Fig 5(d-f)), the illumination initially increased and peaked before decreasing with the light transport distance in the positive *x*-direction. From curve a (*y *= 0 m), the illuminance values at the origin were 1776.9, 1358.2, and 810.4 lx at angles of 45°, 60°, and 75°, respectively. Curves d and e show that the illuminance values along *y *= − 5 m and *y *= − 10 m were much lower and showed slight variations with distance, with the maximum illuminance value for each angle occurring only in the range of 2–30 lx.

**Fig 6:**
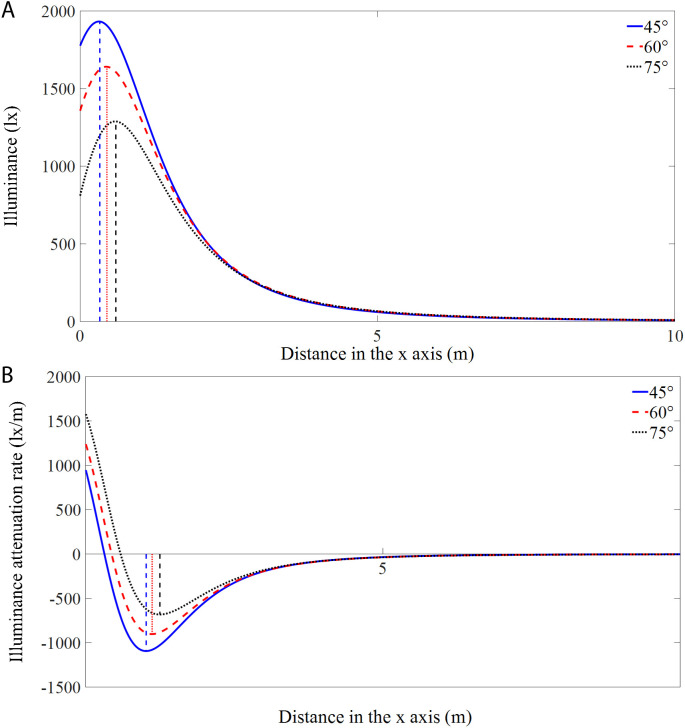
Illuminations (a) and attenuation rates (b) of the RVL along the axis of y = 0 m at different angles.

Fig 6 shows the illumination variations and the corresponding attenuation rates with distance along the axis of *y* = 0 m of the RVL at different angles. As presented in Fig 6(a), the maximum illuminance value decreased with increasing angle, while the distance from the origin to the position of maximum illuminance increased. Fig 6(b) shows that the variations in the illumination attenuation rate (IAR, refers to the rate at which the light intensity decreases when it propagates from the light source through the medium) with distance exhibit positive and negative phases, and the positions of the maximum IAR vary at different angles. The absolute value of IAR first decreased, then increased, and finally decreased to 0 lx/m with a further increase in distance. In the positive phase, the IAR increased as the angle increased, while in the negative phase, the absolute value of the IAR decreased with an increasing angle. Specifically, the absolute value of the maximum IAR was 1093.5 lx/m at a distance of 1.02 m from the origin when the module was at an angle of 45°. The maximum IAR located at the origin was 1239.0 lx/m at an angle of 60°, and the maximum IAR located at the origin reached 1578.1 lx/m when the angle was 75°.

#### The rectangular horizontally mounted lamp (RHL).

**Fig 7:**
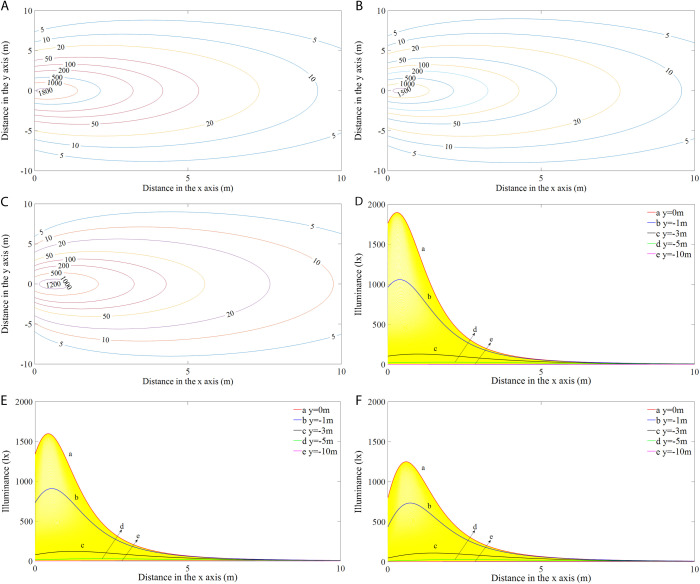
Illumination distributions (a-c) and variations in illuminance values along the x-axis (d-f) of the RHL at different angles.

Fig 7 shows the illumination distributions (left) and the illuminance variations for values of *y* = 0, −1, −3, −5, and −10 m along the positive *x*-axis direction (right) of the RHL at different angles. The illumination distributions (Fig 7(a-c)) and illuminance variations (Fig 7(d-f)) showed similar patterns when the modules were mounted horizontally and vertically, except that the maximum illuminance values and their distances from the origin were different. The isolines were denser in the region below the light source and were mainly distributed within a range of 3 m from the light source. From curve a, the illuminance value at the origin was 1763.2 lx at an inclination angle of 45°, while at 60° and 75°, they were 1342.3 and 798.5 lx, respectively. Curve e shows that the illuminance values at *y* = −10 m remained almost constant for all angles and did not change with distance.

**Fig 8:**
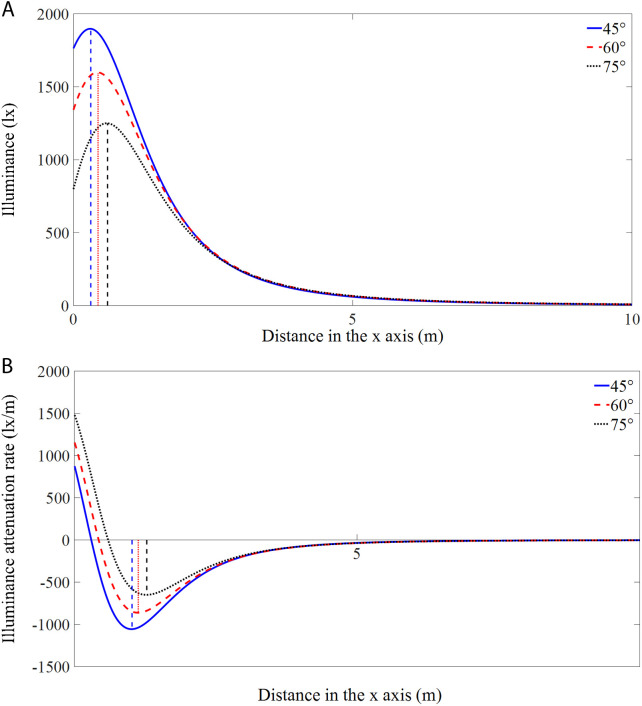
Illuminations (a) and attenuation rates (b) of the RHL along the axis of y = 0 m at different angles.

Fig 8(a) shows the illumination variations with distance along the axis of *y* = 0 m direction of the RHL at different angles. As the angle increased from 45° to 60° to 75°, moderate decreases in the maximum illuminance were observed, but the distances from the origin to the location of the maximum illuminance value increased. The IAR variations with distance are displayed in Fig 8(b). Specifically, the absolute value of the maximum IAR was 1056.0 lx/m at a distance of 1.01 m from the origin at an angle of 45°, and the maximum IARs located at the origin were 1154.7 and 1480.6 lx/m when the modules were angled at 60° and 75°, respectively.

#### Circular lamp (CL).

**Fig 9:**
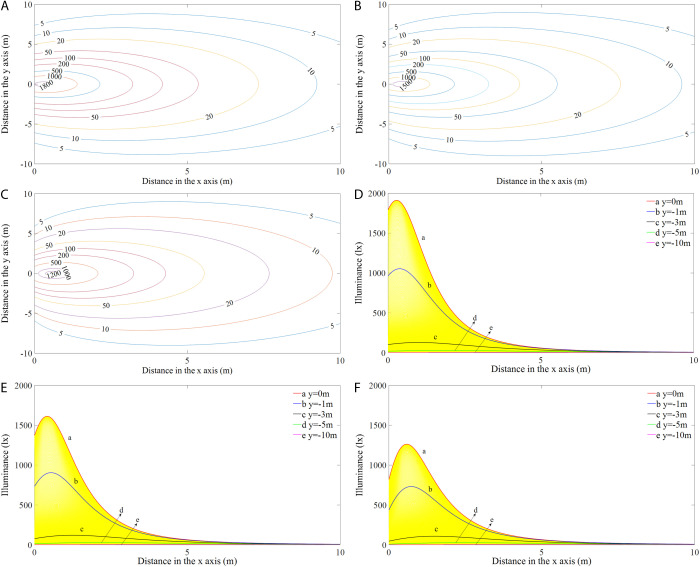
Illumination distributions (a-c) and variations in illuminance values along the x-axis (d-f) of the CL at different angles.

Fig 9 shows the illumination distributions (left) and variations in illuminance values for *y* = 0, −1, −3, −5, and −10 m in the positive *x*-axis direction (right) for the CL at different angles. Similarly, the characteristics of the illumination distribution for the CL are similar to those of the RVL and RHL for each angle (Fig 9(a-c)), but there were differences in the maximum illuminance values and their distances from the origin under different configurations (Table 2). The maximum illuminance values for different shaped lamps at the same angle vary considerably, with the RVL having the highest value, followed by the CL, and the RHL having the lowest illuminance value. The locations of maximum illuminance for each module type are relatively close to each other. From curve a (Fig 9(d-f)), the illuminance values at the origin were 1792.8, 1372.7, and 820.0 lx, corresponding to angles of 45°, 60°, and 75°, respectively.

**Table 2:** The maximum illuminance values (lx) and distances to the origin (m) of various configurations at different angles.

Angle Configuration	45°	60°	75°
RVL	1932.6 (0.33)	1641.4 (0.45)	1289.0 (0.60)
RHL	1897.9 (0.31)	1598.7 (0.44)	1250.4 (0.60)
CL	1912.5 (0.29)	1613.2 (0.42)	1263.4 (0.59)

The distances from the origin and the positions of the illumination curves for 1000 and 10 lx for the three module types at different angles are summarized in Table 3. A comparison of the position of the two illuminance values indicated that the illumination distribution of the RVL in the light field was different from those of the RHL and CL, especially for the small installation angle. Taking the RVL as an example, when the module had an angle of 45°, the distances from the origin to 1000 and 10 lx were 1.42 and 9.22 m, respectively. When the angle increased to 60°, the distance from the origin to 1000 lx was 1.37 m and to 10 lx was 9.58 m. At an angle of 75°, the distances from the origin to 1000 and 10 lx were 1.22 and 9.74 m, respectively.

**Table 3:** Distances (m) from the origin for illumination curves of 1000 and 10 lx of three module types at different angles.

Illuminance Configuration	1000			10		
	45°	60°	75°	45°	60°	75°
RVL	1.42	1.37	1.22	9.22	9.58	9.74
RHL	1.40	1.35	1.19	9.24	9.59	9.75
CL	1.40	1.35	1.20	9.25	9.59	9.75

**Fig 10:**
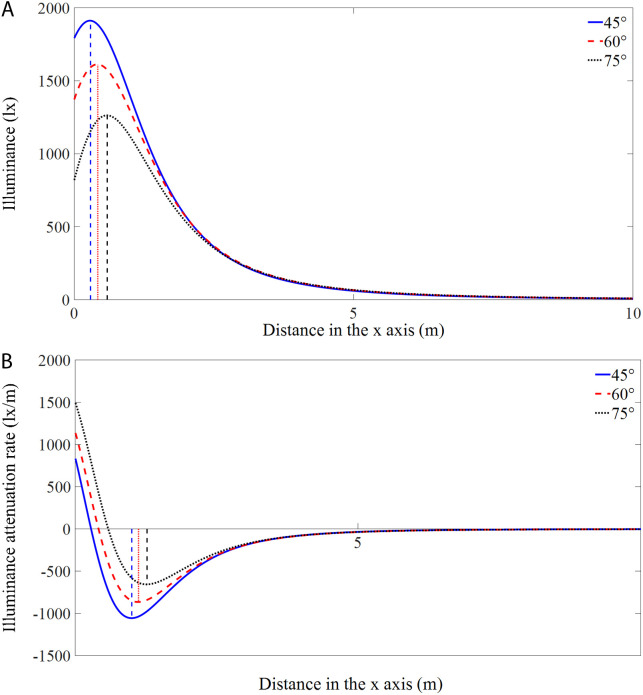
Illuminations (a) and attenuation rates (b) of the CL along the axis of y = 0 m at different angles.

Fig 10 shows the illumination variations and IARs with distance along the axis of *y* = 0 m of the CL at different angles. Overall, the illumination changes for the CL are similar to those of the vertically and horizontally mounted rectangular lamps. Specifically, at an angle of 45°, the IAR at 0 m is 832.7 lx/m, with a maximum value of −1056.2 lx/m at a distance of 1.0 m. At 60° and 75°, the IAR at 0 m is maximized with values of 1136.2 and 1492.0 lx/m, respectively.

### Differences in the lighting characteristics of lamps with different configurations

Based on the simulated illumination data, the differences in illumination distribution characteristics among RVL, RHL, and CL were calculated for different configurations. The difference in illuminance value is obtained by:


$$E_{1-x}=E_{1}-E_{x}$$ (6)

Where $E_{1}$ is the illumination of the RVL; $E_{x}$ (*x* = 2, 3) represents the illuminance values of the RHL and CL, respectively; and $E_{1-x}$ is the illuminance difference between the RVL and the other two lamp types.

#### Illuminances differences between vertically and horizontally mounted rectangular lamp (RVL and RHL).

**Fig 11:**
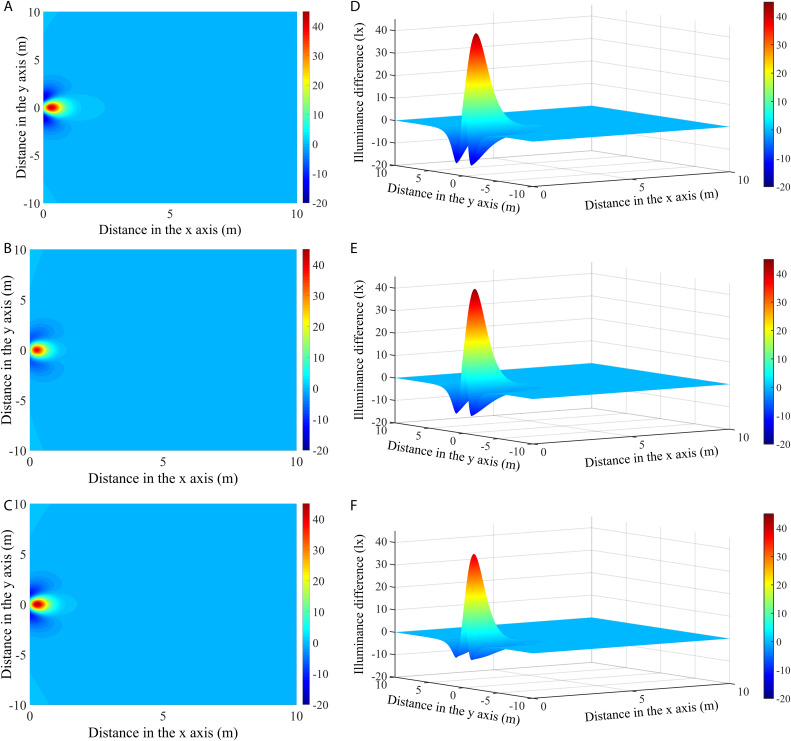
The differences in illumination (a-c) and their three-dimensional distributions (d-f) between RVL and RHL at different angles.

Fig 11(a-c) shows the differences in illumination distributions between vertically and horizontally mounted rectangular lamps (RVL and RHL) at angles of 45°, 60° and 75°. Notably, large illuminance differences in the light field were found within 2 m of the modules, while the other illuminated areas had nearly equivalent illumination levels. To clearly display the dynamics and disparities in illumination among the different configurations, the three-dimensional (3-D) distributions of the illuminance differences are presented in Fig 11(d-f). The visualization shows prominent peaks located directly below the light source (i.e., positive illuminance difference) and recessed areas on either side (negative illuminance difference). In addition, the magnitudes and positions of the illuminance differences between RVL and RHL vary with angle. As the installation angle increased, the positive illuminance difference first increased and then decreased, while the negative illuminance difference gradually decreased. Specifically, when the module was oriented at an angle of 45°, the maximum positive illuminance difference was 43.15 lx at 0.63 m from the origin, and the maximum negative difference was −14.86 lx. At an angle of 60°, the maximum positive illuminance difference was 44.01 lx at 0.56 m from the origin, and the maximum negative difference was −11.75 lx. When the angle increased to 75°, the maximum positive illuminance difference was 39.23 lx at a distance of 0.52 m, and the maximum negative difference was −7.21 lx. The location of the maximum illuminance difference was closer to the light source as the inclination angle increased.

#### Illuminance differences between a rectangular vertically mounted lamp (RVL) and circular lamp (CL).

**Fig 12:**
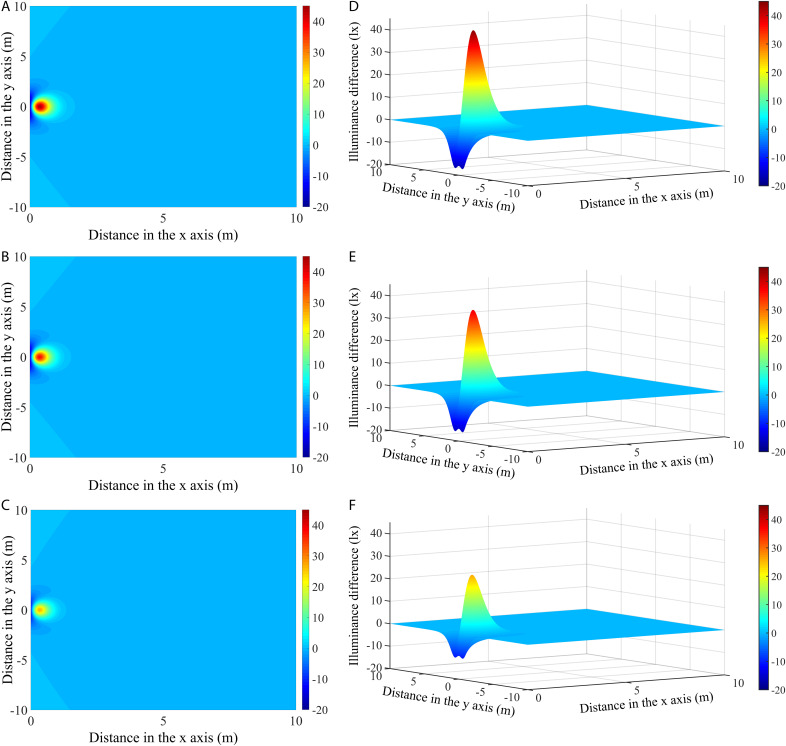
The differences in illumination (a-c) and their three-dimensional distributions (d-f) between RVL and CL at different angles.

Fig 12(a-c) shows the differences in illumination distributions between a rectangular lamp mounted vertically and the circular lamps (RVL and CL) at different angles. As can be seen, the illumination distributions differed significantly among the angles, especially in areas close to the light source. The corresponding 3-D distributions of the illuminance differences are displayed in Fig 12(d-f). Similarly, the RVL had higher illuminance values directly below the light source compared to the CL, and there were negative illuminance differences in the areas on either side of the light source. In addition, it can be seen from the graph that the convex part seemed to decrease more obviously, and the recessed area was sharper. At an angle of 45°, the maximum negative difference was −17.08 lx, and at 60° and 75°, the maximum negative differences were −15.84 and −10.64 lx, respectively. Table 4 tabulates the maximum positive illuminance differences and their distances from the origin of the three lamp types at different angles. The maximum positive illuminance differences were 44.09, 37.98, and 26.04 lx, which correspond to the angles of 45°, 60°, and 75°, respectively. It can be concluded that the positive and negative illuminance differences for both RVL and CL decreased as the angle increased.

**Table 4:** The maximum differences in positive illuminance (lx) and their distances to the origin (m) among various configurations at different angles.

Angle Configuration	45°	60°	75°
RVL and RHL	43.15 (0.63)	44.01 (0.56)	39.23 (0.52)
RVL and CL	44.09 (0.74)	37.98 (0.73)	26.04 (0.68)

## Discussion

### Effects of module shape and mounting mode on lighting performance

The simulation results showed that the RVL, RHL, and CL generally had similar illumination patterns when installed at the same angle, with the iso-illuminance curves distributed symmetrically relative to the axis of *y *= 0 m in the light field. These characteristics are consistent with the light distributions of LED lamps utilized in squid fishing [37]. Specifically, however, noticeable differences in light intensity were found among the three modules, particularly in the area surrounding the light source. Additionally, the locations of the 10 and 1000 lx illuminance curves for lamps with different shapes and mounting modes were close to each other (Table 3), further indicating that the illumination distributions of these various light sources mainly differed around the artificial light.

Light intensity and the illumination uniformity in underwater illuminated zones are key factors that affect the aggregation and successful capture of phototactic fishes [38,39]. With respect to the differently shaped lamps, similar trends of IAR variations were observed due to their comparable illumination distributions. At the same angle, the IAR fluctuated more near the light source, showing a pattern of initial decrease, subsequent increase, and finally decreased again with distance, which was caused by the phenomenon that the maximum illuminance value was not located at the origin in the light field. As illustrated by the *y *= 0 axis, the region around the light spot had higher illuminances in the illuminated zone, with the maximum value located at the center of the spot. Conversely, due to the denser light isolines in the light spot region, the associated variations in IAR were instead small and then increased symmetrically from the spot center as a threshold. With increasing distance from the light source, the IAR gradually decreased after a certain illuminance level was reached. Compared with the IAR values of RHL and CL, the IARs of RVL were slightly larger at the same angle, but there were no significant differences among the IARs of the three types of fishing modules (*P* = 0.98, ANOVA).

The illumination distributions are largely related to the shapes and installation methods of the LED fishing lamps. Comparisons of the illuminance differences among the three types of modules showed significant convexity and concavity, and it is worth noting that the curvatures of the peaks and valleys of the illumination differences varied for the different lamp types (Figs 11 and 12). Interestingly, the illuminance levels in the region directly below the RVL were always higher than those of the RHL and CL, while exhibiting the lowest light intensity on both sides of the light source. Specifically, the 3-D illuminance differences between RVL and RHL depicted sharper and broader shapes, whereas the distributions for CL appeared relatively shorter and narrower. This disparity implied that the illuminance difference values between RVL and RHL were larger than those between RVL and CL (Table 4). This result is due to the narrow width of the rectangular module, where the internal lamp beads resemble a longitudinal strip when mounted vertically. In contrast, when the module is installed horizontally, the illuminance values decrease because most of the beads occupy more space in the horizontal direction, which causes the light beam to be more dispersed over the illuminated region. As for the CL, although it has the same center height as the RVL, the lamp beads are more concentrated in the upper position of the module, with fewer beads in the lower position. Due to the highly directional nature of the LED light, only a small amount of light is scattered to the sides of the lamp, resulting in relatively low illuminance levels in the side zones. In summary, the differences in illuminance between RVL and RHL are most significant in those areas directly below the lamp modules, while the greatest difference with CL occurs in the side areas that are close to the light source.

The effective illuminated water volume is usually considered an indicator to evaluate the fish attracting ability of fishing lights, both in terms of horizontal distance and vertical light transmission depth [40]. Although underwater illumination was not considered in the present work, the magnitude of the illuminance difference could reflect the vertical light transmission depth as well as the lighting capability of the different modules. Fishing lamps with different shapes varied considerably in their illumination levels near the light source, but little distinction was found in the illumination range in the horizontal direction. This situation demonstrates that the lamp shapes primarily affect the penetration depths of the underwater light beams but have a limited effect on the horizontal attraction distances. A greater illumination difference was correlated with a larger vertical attraction depth, which facilitated the attraction of fish from deeper water layers. Our results indicate that using RVLs would produce stronger luminous intensity levels and greater light penetration depth compared to those with RHLs and CLs.

### Effects of module installation angle on lighting performance

The underwater lighting characteristics and behavioral responses of fish are strongly affected by and are highly sensitive to changes in the mounting angle of the lamp pole. An analysis of the illumination levels and distributions of all three lamps with different shapes at angles of 45°, 60°, and 75° revealed a gradual decrease in maximum illuminance with increasing angle, which was accompanied by a simultaneous increase in distance from the origin (Table 2). The reason for this trend is that as the angle increases, more light rays are directed to more distant regions, while the light is absorbed and scattered during transmission, resulting in a constant decrease in maximum illuminance and a position further away from the light source. Lamp poles with large angles cover a wider illuminated area, with more uniform light intensity distributions and smaller illuminance differences in the light field (Table 4). Xia noted that excessively small or large angles of the fishing lamp boxes would result in poor fish attraction, as the angle plays a significant role in the underwater illumination distributions [41]. The variations in IAR indicated that larger angles corresponded to greater illumination attenuation levels in the vicinity of the light source. Once beyond the location of the maximum illuminance value (where the IAR reached 0), the illumination attenuation continued to decrease due to the variations in illumination characteristics with distance and the relatively low light intensities in remote regions. This observation is consistent with the findings of Hua et al., who found that smaller angles corresponded to larger fluctuations in illumination within the light field [27], similar to the variations in light attenuation that were observed beyond the location of the maximum illuminance value in this study.

Fishing lamp system with reasonable configuration can be helpful to increase effective lighting volume, reduce fuel consumption and carbon dioxide emissions, regulate the total light power of fishing lights and promote the sustainable management of light fisheries. Given the differences in the illumination characteristics of the LED modules at different mounting angles, it is essential to determine a reasonable lamp configuration to effectively attract fish. This factor is especially important when considering the large number of lamp poles on a saury fishing vessel (approximately 127 poles or 508 modules) and the low visual threshold needed for habitual aggregation of saury (10^−2^ − 10^−5^ 1x) [42]. In the case of RVLs, for example, the distances from the origin of the 10 and 1000 lx iso-illuminance curves were 7.80, 8.21, and 8.52 m at angles of 45°, 60°, and 75°, respectively (Table 3). This finding indicates that the horizontal attraction distance of identically shaped lamps increases with angle, but the rate of increase gradually decreases. Too large an angle not only tends to decreases in the attraction depth and light utilization efficiency but also increases the light attenuation and wind resistance of the lamps in the air [43], and most of the light energy would be wasted when the angle reaches 90°. Inada and Ogura reported that large amounts of light are reflected when the lamp poles are installed at angles of more than 70° [44]. In addition, adjusting the installation angles of the lamp poles could affect the illumination characteristics of the lighting equipment and thus the location of fish aggregation around the fishing vessel. To some extent, maintaining an appropriate distance between the illuminated waters for schooling fish and the vessel facilitates the operation of fishing gear by the fishermen on deck [38]. Based on these considerations, we recommend that the optimal installation angle for LED fishing lamps used for saury fishing is 60° to 75°. Similarly, Jeong’s research concluded that the angles for LED fishing lamps used for squid fishing should be between 60° and 75° based on a comparison of the illumination distributions of metal halide lamps and LEDs [45].

## Conclusions

Understanding the correlation between illumination characteristics and artificial light configurations is crucial for rationalizing onboard lighting systems. This study proposed an engineering solution for regulating and controlling the total light power of fishing lamps using a configuration optimization approach. The illumination performances of three types of commercial fishing lamps (RVL, RHL, and CL) installed at different angles were evaluated by means of numerical simulation. The results confirm that the shape and installation forms of fishing lights have significant effects on the illuminated field, and the artificial lamp manufactured into rectangular shape and mounted vertically at installation angles of 60° to 75° had superior effects in attracting saury. Further integrated analyses of underwater illumination, structure of fishing vessel, phototaxis characteristics of fish, and other configuration parameters associated with fish aggregation patterns are needed to improve the understanding of light fishing mechanisms, and more effective efforts should be devoted to optimizing the configurations of artificial light to reduce light power competition and fuel consumption.

## Supporting information

S1 File The fitted illumination data of LED lamp at different angles. (7Z)
